# The Survey of the Health of Wisconsin (SHOW), a novel infrastructure for population health research: rationale and methods

**DOI:** 10.1186/1471-2458-10-785

**Published:** 2010-12-23

**Authors:** F Javier Nieto, Paul E Peppard, Corinne D Engelman, Jane A McElroy, Loren W Galvao, Elliot M Friedman, Andrew J Bersch, Kristen C Malecki

**Affiliations:** 1 Department of Population Health Sciences, University of Wisconsin School of Medicine and Public Health, Madison, Wisconsin, 53726, USA; 2 Center for Urban Population Health, Milwaukee, Wisconsin, 53233, USA; 3 Department of Family and Community Medicine, University of Missouri School of Medicine, Columbia, Missouri, 65212, USA

## Abstract

**Background:**

Evidence-based public health requires the existence of reliable information systems for priority setting and evaluation of interventions. Existing data systems in the United States are either too crude (e.g., vital statistics), rely on administrative data (e.g., Medicare) or, because of their national scope (e.g., NHANES), lack the discriminatory power to assess specific needs and to evaluate community health activities at the state and local level. This manuscript describes the rationale and methods of the Survey of the Health of Wisconsin (SHOW), a novel infrastructure for population health research.

**Methods/Design:**

The program consists of a series of independent annual surveys gathering health-related data on representative samples of state residents and communities. Two-stage cluster sampling is used to select households and recruit approximately 800-1,000 adult participants (21-74 years old) each year. Recruitment and initial interviews are done at the household; additional interviews and physical exams are conducted at permanent or mobile examination centers. Individual survey data include physical, mental, and oral health history, health literacy, demographics, behavioral, lifestyle, occupational, and household characteristics as well as health care access and utilization. The physical exam includes blood pressure, anthropometry, bioimpedance, spirometry, urine collection and blood draws. Serum, plasma, and buffy coats (for DNA extraction) are stored in a biorepository for future studies. Every household is geocoded for linkage with existing contextual data including community level measures of the social and physical environment; local neighborhood characteristics are also recorded using an audit tool. Participants are re-contacted bi-annually by phone for health history updates.

**Discussion:**

SHOW generates data to assess health disparities across state communities as well as trends on prevalence of health outcomes and determinants. SHOW also serves as a platform for ancillary epidemiologic studies and for studies to evaluate the effect of community-specific interventions. It addresses key gaps in our current data resources and increases capacity for etiologic, applied and translational population health research. It is hoped that this program will serve as a model to better support evidence-based public health, facilitate intervention evaluation research, and ultimately help improve health throughout the state and nation.

## Background

Health is fundamentally determined by the social, physical, economic, and political environments, in addition to biological and behavioral factors [1,2]. This broad health determinants model is particularly relevant for understanding the distribution and developing the means for prevention of some of the most prevalent chronic conditions (e.g., cardiovascular disease, diabetes, obesity, psychiatric disorders) [3]. In this context, high quality and comprehensive data systems that take into consideration the complex interaction of both individual, community, and contextual determinants are important for the identification of health needs, for the systematic assessment of health inequalities, and for the evaluation of the impact of policies and programs. Whereas available national surveys provide high-quality data of the nation's health and trends in health outcomes and their determinants, they lack discriminatory power to assess the health of local communities where community health interventions usually occur.

This paper describes the design of and rationale for the Survey of the Health of Wisconsin (SHOW), a novel statewide infrastructure for population health data collection that was established in Wisconsin in 2008. Annual surveys of a representative sample of state residents include individual interviews, a physical exam and biospecimen collection, coupled with assessments of the community environment. In addition, SHOW supports the establishment of basic and applied population health research programs, i.e., by providing the foundation for ancillary study initiatives addressing a diverse set of statewide and local population health inquiries.

The SHOW and initial ancillary studies described here build on growing population health data systems, resources, and institutional commitment at the University of Wisconsin School of Medicine and Public Health. The program aspires to serve as a new model for applied population health research at the state, local (e.g., county), and community levels.

## Methods/Design

### Overview

The program is built on a broad determinants of population health model [4]. It addresses health data about individuals (including biological, demographic, psychosocial, and behavioral), their family (e.g., socioeconomics, household physical characteristics), and their community (e.g., physical, social and built environment, health care quality).

The SHOW uses "state-of-the-art" methodology on annual representative samples of the Wisconsin civilian, non-institutionalized adult population with a longitudinal follow-up component. The main survey is comprised of a data collection core organized in a modular structure that allows the program to: adapt to evolving research priorities; be used as a tool for the evaluation of statewide or community-specific public health interventions; and serve as a foundation for the addition of epidemiologic and community health ancillary studies.

The specific aims of the program are: 1) to provide an infrastructure for ongoing and future health sciences research; 2) to improve monitoring and enable promotion of health for all Wisconsin residents; and 3) to facilitate innovative and transformational population health research. The program creates a basis for research about population health outcomes and determinants, thus helping monitor the state and the national Healthy People objectives [5].

Funding for the planning and implementation of SHOW was provided by the Wisconsin Partnership Program (WPP), an endowment established by the University of Wisconsin (UW) Medical School in 2003 with proceeds from the conversion of BlueCross/BlueShield of Wisconsin into a for-profit corporation. WPP received the explicit directive that these funds were to be used to improve the health of the state population. As the program grew, additional funding from the National Institutes of Health, National Heart, Lung, and Blood Institute and other extra- and intra-mural sources was obtained.

The SHOW protocol and informed consent documents are approved by the UW-Madison Health Sciences Institutional Review Board. Additionally, data are collected under a Certificate of Confidentiality obtained from the US Department of Health and Human Services.

This section summarizes the design features of the SHOW; further detail can be obtained from the Survey Protocol and Manual of Operations downloadable from http://www.show.wisc.edu/protocol.

### Target population and recruitment goals

The SHOW sampling frame is Wisconsin non-institutionalized/non-active duty adult residents ages 21-74 at the time of initial selection (longitudinal evaluation follows subjects beyond age 74). However, all household members (including children and older adults) are enumerated (see below), and may be the focus of targeted ancillary studies.

Presently, the goal is to continue this survey indefinitely, recruiting an independent sample of 800-1,000 survey participants each year. This target sample size is driven by a broad range of potential outcomes and research questions to be addressed with SHOW and in anticipation that researchers will use these data in multiple ways with varying sample size requirements.

### Sampling procedures

**Figure 1:**
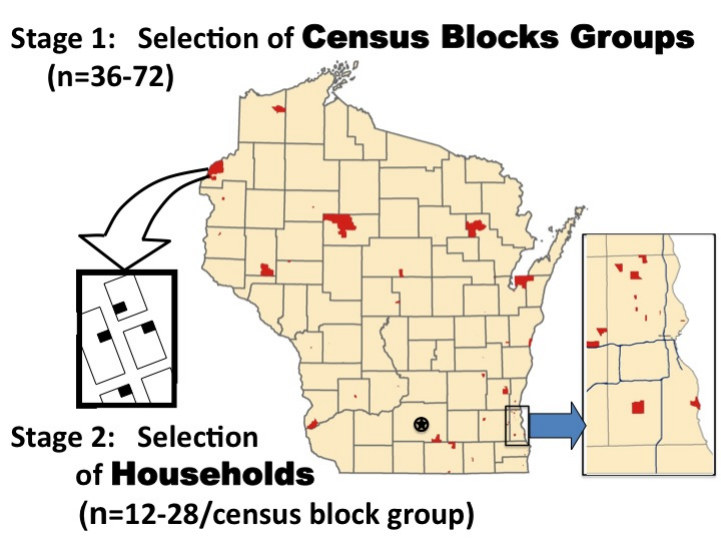
**SHOW's two-stage sampling design**. Census block groups (CBGs) in urban areas such as Madison (marked with a star) and Milwaukee (in enlarged inset) cover a smaller area due to increased population density and are barely visible in this map.

Every year, survey participants are selected from a random sample of households using a two-stage, probability-based cluster sampling approach (see Figure 1):

#### Stage 1

The initial sampling frame is constructed using Census 2000 data to generate 4,388 Census Block Groups (CBGs) or clusters of CBGs for use as the primary sampling units (PSUs). CBGs that fall entirely in sovereign Indian nation territories from tribes that have not yet provided explicit authorization to be included in the survey are excluded (n = 13). CBGs with less than forty households are merged with a neighboring CBG to form "cluster" sampling units. In order to ensure a representative distribution of the sample across the entire spatial and sociodemographic range of the state population, the PSUs are stratified according to two criteria: 1) congressional district (8 strata); and 2) percentage of the population living below 100% of poverty level. Sampford explicit probabilities proportional to size without replacement sampling method [6] is used to randomly select CBG. Since the initiation of the survey, the number of sampled CBGs per year has varied between 36 and 72 depending on logistics and personnel considerations.

#### Stage 2

SHOW uses a variety of up-to-date data resources and geographic information system (GIS) technology to create its household sampling frame. A list of household addresses by CBG is generated using commercially available United States Postal Service (USPS) Delivery Sequence Files purchased from MSG-Genesys (Marketing Systems Group, Fort Washington, PA). These delivery sequence files use zip + 4 Topologically Integrated Geographic Encoding and Referencing (TIGER) System files to link USPS addresses to CBGs.

As described elsewhere in detail http://www.show.wisc.edu/protocol, GIS and extant data including Google Earth, publicly available county maps http://coastal.lic.wisc.edu/wisconsin-ims/wisconsin-ims.htm, and digital tax assessment data from each county are then used to clean and enhance MSG-Genesys files according to a defined protocol.

From the household sampling frame, 12-28 addresses are randomly selected using simple random sampling. The targeted number of households by CBG is held constant within an annual cycle but this number may be adjusted annually as experience provides better direction regarding the range of response rates in the different areas of the state. As a final quality assurance step in the household sampling frame development, the field teams conduct a modified *half-open interval *procedure to identify households that may have been missed in the original sampling frame [7].

### Recruitment methods

Recruitment of SHOW participants begins with in-person contact by study staff at the selected household address. Prior to initial contact, no information is known about the household residents. An effort is made during the recruitment process to enumerate all household members and enroll eligible individuals to participate in the survey.

#### Enhancing Community Awareness

To ensure public awareness and increase participation, a public relations campaign is launched in communities (defined as major cities or jurisdictions intersecting a selected CBG) six to eight weeks before recruitment in that location. The campaign includes phone contact with and/or an introductory letter sent to local public officials and both formal and informal community leaders to notify them of the timeframe for household recruitment in their area and request their verbal and written endorsement of the study. These endorsement letters are shown to individuals being recruited to verify legitimacy of the study and enhance participation. Posters and flyers are distributed for display in health care centers, public schools, churches, libraries, and businesses. Local newspapers, television, and radio stations are also contacted, and press releases provided.

#### Household and Individual Recruitment Approach

One to three weeks before a SHOW team arrives in a selected block group, the randomly selected households are mailed a package containing endorsement letters and an introductory letter (addressed to "Resident") that describes the project, explains how their household was selected, lists the benefits of participation, and explains that a SHOW field team member will be knocking on their door in the next few weeks. Mailed materials are provided in English but are also made available in Spanish during initial household recruitment in predominantly Spanish-speaking households.

If there is no response on the first visit to a household, informational materials, including a description of SHOW and contact information, are left at the door. Subsequently, up to six home visits, at different times of the day and different days of the week, are attempted in order to gain contact with each selected household.

At the initial person-to-person contact, the field team verifies that the respondent is at least 18 years old, introduces the study, and asks to proceed with screening. If necessary, flexible times (including evening and weekend hours) are offered for staff to return to the household to complete the screening process. The screening instrument is used to enumerate all household members according to gender and age and determine eligibility. An attempt is made to obtain a response to these questions even for individuals who decline participation in the survey. Names and contact information are collected for all eligible participants.

Individuals must meet all of the following inclusion criteria for participation in the survey: 1) the selected household is their usual place of residence (defined as anticipated residence at this address for more than 6 months during the current calendar year); 2) age 21-74 years; 3) mentally capable of giving written informed consent; and 4) able to communicate answers to interview questions.

Individuals are *excluded *if they are: 1) residents of nursing homes, hospitals, mental institutions, penal institutions, jails, halfway houses, college dormitories, or are under the jurisdiction of the Department of Corrections; 2) fulltime members of the armed forces or activated units of the National Guard who are currently stationed away from home; 3) persons who have multiple residences and who spend less than half their nights in the current year at the selected residence (including students living away from their primary residence); and 4) residents who voluntarily disclose a diagnosis of mental incapacity and there is no representative available to be a proxy respondent.

After the screening process, eligible household members are informed of the scope of the survey, time commitment, possible risks and benefits, and are invited to participate. There is no requirement that all eligible members of the household participate. Recruited participants are also informed that they can refuse to answer any survey questions and decline to participate in any or all of the physical exam or biospecimen collection. Incentives for participation include a t-shirt with the project's logo, an individualized findings report with selected results of physical exams and blood tests (see below), and up to $95 in compensation for selected survey instrument completion and biospecimen collection, plus travel and child care expenses.

### Survey interviews and physical exam

Data collection is divided into three major components: an in-home interview (Time 1); a self-administered questionnaire (Time 2); and a mobile exam center or fixed clinic visit that includes a physical exam, biospecimen collection, and more personal data collection (Time 3).

The survey at Time 1 is administered at the home of consenting participants and includes topics listed in Table 1. The computer assisted personal interview (CAPI) lasts about one hour. Additional tracking information is also collected and includes name, social security number (or last 4 digits), address, telephone numbers (including mobile phone), email, and mailing address of at least two friends or relatives.

**Table 1:** Survey visits and survey components.

SHOW Visit	Topics Covered	
**Time 1**: **Interviews **(most computer-assisted) conducted in households following eligibility determination and informed consent	• Tracking information	• Sensory health (hearing, vision)
	• Demographics and occupational history	• Dental health
	• Housing characteristics	• EuroQol questionnaire (health-related quality of life)
	• Health history (Part 1)	• Health insurance, health care access & utilization
	• Prescription & over the counter medications	• Physical activity, exercise habits
	• Health screening & immunization history	

**Time 2**: **Self-administered questionnaire **left with participants	• Prevention & safety habits	• Sleep habits and problems
	• Dietary habits	• SF-12 (health-related quality of life)
	• Stress, discrimination, life events inventory	• Military experience

**Time 3**: **Interviews, questionnaires, physical exam & biological sampling **conducted at one of four exam centers--2 MECs and 2 fixed-sites (Milwaukee, Middleton)	• Health history (Part 2)	• Cognitive function
	• Weight history	• Health literacy
	• Women's reproductive health history, contraceptive use	• Weight; height; waist, hip & arm circumference
	• Mental health screener; depression, post-traumatic stress disorder inventories	• Bioimpedance analysis
	• Smoking and alcohol habits	• Sitting blood pressure and pulse
	• Food security	• Peak flow meter (respiratory function)
		• Phlebotomy and urine collection

Upon completion of the Time 1 survey, a booklet with additional questionnaires to be self-administered (Time 2) is left with each participant (see topics in Table 1). Finally, a visit to a SHOW exam center (Time 3) is scheduled. Time 3 appointments are usually made within 1-2 weeks of the initial data collection (at Time 1) and consist of additional CAPI interviews, audio-assisted computer self-interviews (ACASI), and a physical exam including anthropometry, blood pressure, and body fat composition. Blood (or saliva) and urine specimens are also obtained. Table 1 lists topics included during Time 3 surveys.

**Figure 2:**
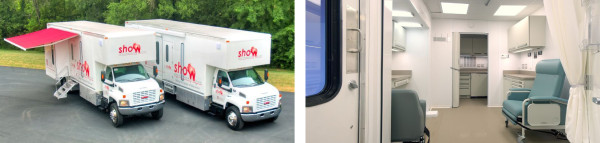
**SHOW's mobile exam centers**. Interior of the mobile exam center shown in the right hand side.

Appointments are scheduled in various clinic sites across the state. There are two permanent clinic sites (one in Middleton, Dane County, and one in downtown Milwaukee) that participants within a 30-45 mile radius of the centers are asked to visit. In addition, the University of Wisconsin has partnered with the Marshfield Clinic Research Foundation to conduct Time 3 visits in fixed outpatient clinic sites in northern Wisconsin. Two mobile exam centers (34-foot trucks built to specifications by LifeLine Mobile Inc., Columbus, OH--including exam rooms and a mini-laboratory for sample processing) travel to collect data in communities more remote from the permanent sites (Figure 2).

#### Physical Examination and Specimen Collection

Pulse and blood pressure are measured after a five-minute rest period in a sitting position. Blood pressure is measured using an OMRON IntelliSense^® ^Blood Pressure Monitor, Model HEM-907XL (Bannockburn, IL) following recommended procedures [8]. Three systolic and diastolic measurements (5^th ^Korotkoff sound) are taken, with one minute between measurements. Pre-determined systolic and diastolic cut points determine if the participant requires immediate care and emergency services need to be contacted.

Pulmonary function is measured using an electronic peak flow meter (Jaeger AM, Yorba Linda, CA), a validated instrument [9]. The study participant is asked to take a deep breath and exhale as quickly and completely as possible through the tube connected to the measuring device. The test is repeated up to eight times and the highest forced expiratory volume in one second (FEV1) and forced vital capacity (FVC) are recorded.

Bioelectrical impedance analysis (BIA) is conducted using a Quantum X Analyzer (RJL Systems, Clinton Township, MI) to estimate body composition (total body water and body fat). The subject lies on an exam table and has electrodes applied to one arm and one leg.

Anthropometric measurements include: 1) body height (measured to the nearest half centimeter, positioning the head according to the Frankfurt plane and using a SECA 222 wall-mounted stadiometer--SECA Corp., Hanover, MD); 2) body weight (measured to the nearest 100 gr, using a digital Health-O-Meter 725KL--Sunbeam Products, Bridgeview, IL); 3) waist circumference (measured twice at the uppermost lateral border of the ilium using a medical retracting measuring tape); and 4) hip circumference (measured twice with the tape placed below the ileac crest and at the widest point around the buttocks when viewed from the side).

Up to 50 ml of whole blood are collected, aliquoted, and processed immediately at the permanent or mobile exam center. One 5 ml gold top SST tube (Vacutainer, Becton Dickinson Co, Franklin Lakes, NJ) is collected for blood chemistry. This blood is used to extract serum which is then picked up by courier within 48 hours after phlebotomy and transported on dry ice to the Marshfield Clinic Research Foundation Laboratory for measuring blood cell count, serum glucose, serum total and high density lipoprotein (HDL) cholesterol, serum creatinine, and serum glycosylated hemoglobin (hemoglobin A1c). Two 10 ml red top tubes of whole blood are collected for processing and extracting serum for long-term storage in the SHOW Biorepository. Two additional 10 ml Vacutainer lavender tubes are collected for extracting plasma for long-term storage in the biorepository; these tubes are also processed so that the remaining buffy coat and red cells can be stored and batch shipped to Prevention Genetics (Marshfield, WI) for DNA extraction and storage. The serum and plasma to be used for the biorepository are processed in the laboratories and pipetted into 0.5 ml cryovials which are frozen at -80°C. (Samples collected at the mobile exam centers are temporarily frozen at -20°C in the truck's freezer until shipped or transferred to the permanent freezers.) Samples are tracked via the bar-coded labeling system. Participants are not required to be fasting for blood collection, but fasting status is recorded.

Participants who refuse phlebotomy are asked to consent to saliva collection for DNA extraction; this sample is obtained using an Oragene DNA sample collection kit (DNA Genotek Inc., Ottawa, ON, Canada). A spot urine sample is collected from each subject at some point during Time 3.

A few weeks after the exam, a letter describing the results of the physical exam and blood sample values is sent to the study participants; values considered outside normal clinical values are highlighted and participants are instructed to consult with their physician. Blood cell counts and blood pressure values that are below or above certain critical values as specified in the study protocol prompt an immediate recommendation to seek medical care.

### Assessing the quality of the social, physical, and built environment

Numerous extant databases including the US Census, state level air quality, water quality, traffic density, and urban rural classification [based on Rural Urban Commuting Area (RUCA) codes] are also gathered as baseline contextual measures for each CBG. In addition, an objective audit tool, the Wisconsin Assessment of the Social and Built Environment (WASABE), is used to measure key physical, social, and built environment domains in 400 meter street networked buffers surrounding each participant's household. The development of the WASABE audit tool was based on previously validated instruments [10,11], adapted for use in both rural and urban environments in Wisconsin.

### Quality assurance and quality control procedures

Detailed protocols for all aspects of recruitment and data collection have been developed (available at http://www.show.wisc.edu/protocol). Field staff are centrally trained and certified. Digit controls and checks have been programmed into the CAPI and ACASI systems to improve data accuracy.

Interviews are audiotaped (unless refused by the study participant). Randomly selected tapes are reviewed by program staff on an ongoing basis to monitor interviewer adherence to the protocol and instructions.

An extra 3 ml tube of whole blood is drawn from 5% of participants, selected randomly. The blood is aliquoted into phantom duplicate samples for the study of within-person and within-laboratory reliability.

### Analysis plan: cross-sectional and longitudinal components

**Figure 3:**
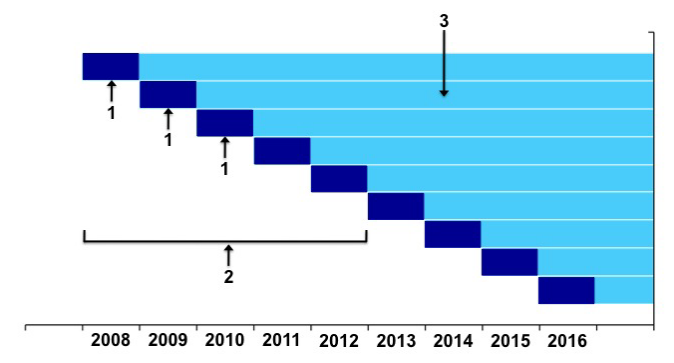
**SHOW's analytical plan: 1) annual cross-sectional surveys; 2) pooling of data from several annual samples (e.g., 2008-2012); 3) person-years for follow-up analyses**.

Each successive annual sample of SHOW participants will be analyzed as an independent sample to assess trends in the prevalence of health outcomes and determinants over time (Figure 3, #1). For cross-sectional epidemiological analyses, including subgroup analyses, data from several annual surveys may be pooled in order to achieve sufficient statistical power (e.g., SHOW 2008-12, Figure 3, #2). Furthermore, participants in successive annual surveys will be accrued into a progressively larger cohort (with staggered entries) that can be followed prospectively for a variety of health outcomes (Figure 3, #3). Follow-up data will be accrued by both passive surveillance (e.g., linkage with National Death Index, hospital discharge data, Medicare databases) and active (bi-annual follow-up calls). Nested case-control or case-cohort analyses [12] for specific outcomes will build on the baseline surveys and person-time of follow-up. Statistical analyses will take into consideration the complex (multistage cluster) design of the survey [13].

### Statistical power

The sample size for this survey (about 800-1,000 participants per year) was determined by a combination of statistical power and feasibility/cost considerations. Given the broad range of objectives to be addressed by this project, a fixed power calculation for the entire SHOW study is not relevant or meaningful. The statistical power will be different for cross-sectional and longitudinal analyses and will vary depending on the frequency of each specific outcome of interest. Moreover, as successive years are accrued, the sample size and statistical power will increase. For ancillary studies (discussed below), the statistical power and the necessary time to accrue sufficient sample size are determined according to each study's specific aims.

### Descriptive characteristics of SHOW participants

SHOW began initial recruitment in June 2008. A total of 616 participants were recruited during the first 18 months of the project (2008-2009) and, as the field operations became more consolidated, 942 participants have been recruited in 2010 (well within the recruitment goal of 800-1,000 per year). While absolute participation rates are hard to compute since the number of eligibles in non-respondent households is unknown, response rates among eligible participants who agreed to be screened was 46% in 2008-2009 and 56% in 2010.

The socio-demographic characteristics of the 2008-2009 SHOW participants are presented in Table 2. The distribution of these variables looks very similar to those obtained in Wisconsin American Community Survey of the US census data (not shown).

**Table 2:** Selected characteristics of the SHOW cohorts, 2008 and 2009 (n = 616*).

Characteristic	Percentage (95% CI)†
Age	
21-39 yrs	37.6 (31.6, 43.7)
40-59 yrs	45.0 (39.2, 50.9)
60-74 yrs	17.3 (13.3, 21.3)

Male gender	50.2 (46.7, 53.7)

Race/ethnicity	
Non-hispanic whites	83.9 (79.1, 88.7)
Non-hispanic African-Americans	6.2 (3.6, 8.7)
Hispanic	3.9 (1.5, 6.3)
Other	6.1 (2.5, 9.6)

Education	
<12 years	6.9 (4.1, 9.7)
12 years	25.3 (21.0, 29.6)
>12 years	67.8 (62.9, 72.7)

Household income	
<25,000/year	21.0 (16.1, 25.9)
25,000-49,999/year	28.2 (23.5, 32.9)
50,000-99,999/year	34.4 (30.1, 38.8)
≥100,000/year	16.3 (10.5, 22.2)

Smoking	
Current	16.3 (12.7, 19.9)
Former	26.8 (22.7, 30.9)
Not allowed in their home	74.1 (70.0, 78.3)

History of physician diagnosed	
Hypertension	28.6 (25.0, 32.2)
Diabetes	9.5 (6.9, 12.1)
Asthma	14.9 (12.9, 17.0)
Cardiovascular disease¶	4.7 (2.8, 6.6)
Depression	6.8 (4.4, 9.2)

Body weight	
Overweight (measured BMI, 25-29.9)	35.3 (29.6, 41.0)
Obese (measured BMI ≥ 30)	38.2 (33.4, 43.0)
Self-reported overweight/obese	68.9 (65.0, 72.9)
Tried to lose weight during last year	58.6 (53.9, 63.4)

Self-reported health	
Very good or excellent	55.0 (49.0, 61.1)
Good	33.5 (28.3, 38.6)
Fair or poor	11.5 (8.2, 14.8)

Health insurance in last year	
Covered, all year	84.4 (81.0, 87.8)
Covered, part of the year	5.9 (3.9, 8.0)
No health insurance	9.7 (7.2, 12.1)

Satisfaction with health care during the last year	
Very good or excellent	78.6 (72.7, 84.6)
Good	17.8 (12.5, 23.2)
Fair or poor	3.6 (1.7, 5.4)

Usually sees the same physician when seeking care	
No	25.5 (21.2, 29.8)
Yes, a family medicine doctor	62.6 (57.1, 68.0)
Yes, an internist	24.0 (19.0, 28.9)
Yes, an obstetrician/gynecologist	5.4 (3.3, 7.5)
Yes, other	8.0 (5.4, 10.7)

Prevention practices	
Women >50 yr with mammography in last year	66.7 (59.1, 74.3)
People >50 yr with colonoscopy in last year	17.7 (13.3, 22.2)
Influenza vaccine in last year	40.6 (35.7, 45.5)

Perception of neighborhood safety from crime for walking or biking	
Very safe	67.5 (61.1, 73.8)
Somewhat safe	29.4 (23.4, 35.3)
Not very safe or not at all safe	3.1 (2.0, 4.3)

	**Mean (Standard Deviation)**

Values from clinical exams	
Body Mass Index (kg/m^2^)	29.5 (8.0)
Systolic blood pressure (mm Hg)¥	123.6 (18.8)
Diastolic blood pressure (mm Hg)¥	77.1 (11.4)
FEV1/FVC	0.83 (0.15)
Total serum cholesterol (mg/dL)‡	192.7 (51.7)
Hemoglobin A1C (%)	5.8 (0.8)

* Number of subjects who completed the in-home portion of the survey. This number is not the number of responses for each characteristic because of missing data in some variables.

† Percentages and confidence intervals are weighted to population characteristics.

¶ History of heart attack, angina, stroke, or transient ischemic attack.

¥ Only people not taking anti-hypertension medication currently.

‡ Only people not taking cholesterol lowering medication currently.

### SHOW as an infrastructure: ancillary studies

One of the core missions of the SHOW is to serve as a platform for the addition of *ancillary studies *that address emerging health-related questions. Table 3 shows examples of SHOW ancillary studies already in progress. These include: 1) studies that require the acquisition of additional data on future or past SHOW participants or the analyses of stored blood samples (e.g., caregiver strain; genetic and environmental determinants of vitamin D levels; oral health exams); 2) studies collecting information on upstream determinants (nutritional environment; county health indicators; quality of care in local hospitals and clinics); 3) studies addressing community-specific population health status before and after community health interventions (e.g., La Crosse and Wood Counties); 4) community-based participatory studies (e.g., community advisory board in Milwaukee); and 5) studies using SHOW and/or mobile clinic resources [e.g., obtaining blood samples for a follow-up study of polybrominated diphenyl ether (PDBE) in Wisconsin fisherman (who were not SHOW participants); validation of drinking water exposure metrics comparing tap water and extant drinking water supply data with urinary biomarkers].

**Table 3:** SHOW ongoing ancillary studies (as of fall 2010).

Study Name	Principal Investigator's Organization/Partner	Target Population	Period	Funding Source	Description
***Studies adding questionnaires/measurements to SHOW participants***					

Caregiver strain and cellular aging	Department of PHS, UW	SHOW participants involved as caregivers	2007-2009	UW Center for Demography of Health & Aging	A questionnaire was added to address caregiver strain and biological samples provided to explore the association between caregiver strain and cellular aging.

Genetic and environmental predictors of serum levels of 25-hydroxyvitamin D	Department of PHS, UW	Random sample of white SHOW participants	2009-2011	Wisconsin Partnership Program - Medical Education Research Committee	Determinants of Vitamin D levels in a subsample of SHOW participants are studied using an additional questionnaire, analyzing blood samples, and assessing of skin pigmentation.

Adult Oral Health Surveillance in Wisconsin	Wisconsin Department of Health Services	All SHOW participants	2010-2011	Department of Health and Centers for Disease Control and Prevention	A brief questionnaire and objective oral health screener exam were added to the core SHOW program to conduct oral health surveillance among Wisconsin adults.

***Studies collecting additional contextual information in SHOW communities***					

Assessing the nutrition environment in Wisconsin communities	Departments of PHS and Nutrition, UW	All SHOW participants	2010-2011	Wisconsin Partnership Program - Oversight and Advisory Committee	Contextual data regarding the availability of nutritional food items in groceries stores and restaurants is being gathered in communities surrounding SHOW participant households.

*Network for Health Equity in Wisconsin*	Department of PHS, UW/UW Population Health Institute/Wisconsin Collaborative for Health Care Quality	All SHOW participants	2009-2012	National Institutes of Health-National Heart Lung and Blood Institute	SHOW individual level data is being integrated into a comprehensive network that will allow for the examination of cardiovascular and respiratory health disparities in the context of Wisconsin communities and provider level quality of care.

***Community-specific health assessments***					

*Communities Putting Prevention to Work *State-Coordinated Small City and Rural Areas - Nutrition and Physical Activity	La Crosse and Wood County Health Department/Wisconsin Department of Health Services	Target Intervention Communities: Counties	2010-2012	Centers for Disease Control and Prevention	SHOW's research infrastructure and resources are being shared as evaluation tools targeted to assess the impact of policy and environmental change interventions to increase physical activity and improve the nutrition environments in Wood County and La Crosse County

***Community Participatory Research***					

SHOW Community Advisory Board: Partners in Dissemination (Milwaukee)	Center for Urban Population Health/Social Development Commission, Milwaukee	African Americans, clients of the Social Development Commission in Milwaukee	2010-2011	Wisconsin Partnership Program	A community-academic partnership to actively involve the lay African American community in Milwaukee in the interpretation of population health data, dissemination of study findings and translation phases of the SHOW.

***Outside studies using SHOW resources***					

Polybrominated Diphenyl Ether (PDBE) exposure from Wisconsin fish	Wisconsin Department of Health Services	Great Lakes Fisher Boat Captains	2008	US Environmental Protection Agency	SHOW infrastructure was used to collect additional biological samples and conduct in-home environmental sampling in a cohort of Wisconsin Great Lakes fisher-boat captains.

Drinking Water Exposure Assessment and Validation for the National Children's Study (Disinfection By-Products)	Department of PHS, UW/National Children's Study/Faculty,	Follow-Up, female SHOW participants of reproductive age	2010-2012	National Institutes of Health - National Children's Study	A sub-sample of women of reproductive age who indicated being on chlorinated municipal drinking water supply will be followed-up; home tap water samples and biomarker data will be collected to ascertain amount of misclassification bias that would result if extant community water supply data alone were used to estimate exposures to disinfection by-products.

UW, University of Wisconsin; PHS, Population Health Sciences.

Ancillary studies must preserve the integrity of SHOW and limit participants' burden, and typically require outside funding. The SHOW policy for ancillary studies can be downloaded from SHOW's website http://www.show.wisc.edu/ancillary.

## Discussion

SHOW's research infrastructure is grounded in a broad population health framework that focuses both on upstream macro- and micro-level health determinants and health outcomes [1,2]. Our model recognizes that health status is highly sensitive to a broad range of determinant factors including biological (e.g., genetic), behavioral (e.g., physical activity, smoking), environmental (e.g., built environment, community policies, air pollution), psychosocial (e.g., stress, social support, exposure to discrimination), socioeconomic (e.g., education, income, occupation), and the quality, availability, and utilization of health care. The applied focus of this program facilitates studies that inform new public health interventions and cost-effective health care planning [4].

SHOW fills a critical gap in applied population health research because it complements existing statewide administrative data (e.g., mortality vital statistics and hospital discharge data) by adding detailed data on health outcomes and determinants both at the individual and at the community level. The finer resolution lacking in national cross-sectional and panel surveys gives SHOW and its ancillary programs (e.g., the Network for Health Equity in Wisconsin, see Table 3) a unique ability to adequately assess health disparities at the community level where multiple determinants operate.

SHOW provides enhanced measurement capabilities not available in other statewide surveys. It uses state-of-the-art household recruitment and individual data collection methods (including face-to-face interviews) to overcome common barriers encountered by traditional phone surveys. SHOW is population-based (not based on clinical populations) and provides a mechanism to collect both subjective (e.g., self-reported history, psychosocial stress, behaviors) and objective individual data (e.g., biological markers, body mass index, blood pressure). This breadth of data collection coupled with contextual community-level data allows for high quality and innovative investigations prospectively exploring interactions between determinants at multiple levels. These resources are necessary in order to truly understand the complexity of competing factors contributing to leading public health problems and finding appropriate solutions [14-16].

Most importantly, SHOW was created as a statewide infrastructure for population health data collection that allows flexibility in addressing national, state, and local community research priorities. The ancillary studies described in the preceding section provide tangible examples of this highly efficient approach to population health research. Another unique strength of SHOW is the scope of research questions that can be addressed. SHOW is built with a modular core providing sufficient flexibility so that ancillary studies can be added as new priorities and funding sources are identified. This could include adding survey components in annual cycles, extending the survey to certain subpopulations, oversampling specific subgroups (i.e., ethnic minorities or geographies), or including children or older adults living in selected households that are currently not eligible for the survey.

Particularly novel is the possibility of using SHOW resources (e.g., equipment, trained personnel, protocols) to carry out community-specific assessments or to evaluate the impact of statewide or local community public health programs, policies, or interventions. These "mini-SHOWs" can be carried out in partnership with local health departments and organizations and are exemplified by the La Crosse-Wood County's *Communities Putting Prevention to Work *initiative (see Table 3). The before and after intervention comparison, coupled with the ongoing annual surveys elsewhere in other communities in the state (*control *communities), will allow an efficient and robust assessment of the effects of the intervention and advance the evidence-based public health agenda.

Another important component of SHOW is dissemination of data and findings not only in academic settings but also at the community level. One goal of disseminating results is to further engage community organizations and health professionals as key recipients of data for use in assessment, program planning, implementation of targeted intervention strategies, and evaluation. The hope is that SHOW will not only be used to find ways to improve the health of Wisconsin residents but also to serve as a unique-in-the-nation model for addressing population health and health disparities by generating community action and facilitating community academic partnerships.

## Abbreviations

ACASI: Audio-assisted Computer Self-Interviews; CAPI: Computer-Assisted Personal Interview; CBG: Census Block Group; DNA: Deoxyribonucleic Acid; FEV1: Forced expiratory volume in one second; FVC: Forced vital capacity; GIS: Geographic Information Systems; NHANES: National Health and Nutrition Examination Survey; PSU: Primary Sampling Unit; RUCA: Rural Urban Commuting Area; TIGER: Topologically Integrated Geographic Encoding and Referencing; SHOW: Survey of the Health of Wisconsin; USPS: United States Postal Service; UW: University of Wisconsin; WASABE: Wisconsin Assessment of the Social and Built Environment; WPP: Wisconsin Partnership Program

## Competing interests

The authors declare that they have no competing interests.

## Authors' contributions

Every one of the authors contributed to the conception of the study, the procurement of funding, the design and coordination, and the preparation of this manuscript. All authors read and approved the final manuscript.

## Pre-publication history

The pre-publication history for this paper can be accessed here:

http://www.biomedcentral.com/1471-2458/10/785/prepub
